# Detection of Rickettsiae, Borreliae, and Ehrlichiae in Ticks Collected from Walker County, Texas, 2017–2018

**DOI:** 10.3390/insects10100315

**Published:** 2019-09-25

**Authors:** Nicole L. Mendell, Erin S. Reynolds, Lucas S. Blanton, Meghan E. Hermance, Andres F. Londoño, Charles E. Hart, Bethany R. Quade, Allen T. Esterly, C’Brionne B. Hendrix, Pete D. Teel, Donald H. Bouyer, Saravanan Thangamani

**Affiliations:** 1 Department of Pathology, University of Texas Medical Branch, 301 University Boulevard, Galveston, TX 77555, USA; nlmendel@utmb.edu; 2 Department of Microbiology and Immunology, SUNY Upstate Medical University, 505 Irving Avenue, Syracuse, NY 13210, USA; ReynolEr@upstate.edu (E.S.R.); hermancm@upstate.edu (M.E.H.); chhart@utmb.edu (C.E.H.); atesterl@utmb.edu (A.T.E.); 3 Department of Internal Medicine, University of Texas Medical Branch, 301 University Boulevard, Galveston, TX 77555, USA; lsblanto@utmb.edu (L.S.B.); bequade@utmb.edu (B.R.Q.); 4 Department of Pathology, Uniformed Services University of the Health Sciences, 4301 Jones Bridge Road, Bethesda, MD 20814, USA; aflondon@utmb.edu; 5 The Institution for Translational Sciences, University of Texas Medical Branch, 301 University Boulevard, Galveston, TX 77555, USA; 6 Department of Cell Biology, University of Texas Medical Branch, 301 University Boulevard, Galveston, TX 77555, USA; cbrhendr@utmb.edu; 7 Department of Entomology, Texas A&M AgriLife Research, College Station, TX 77843, USA; pteel@tamu.edu; 8 Center for Biodefense and Emerging Infectious Diseases, Sealy Institute for Vaccine Studies, Center for Tropical Diseases, University of Texas Medical Branch, 301 University Boulevard, Galveston, TX 77555, USA; 9 SUNY Center for Environmental Health and Medicine, SUNY Upstate Medical University, 505 Irving Avenue, Syracuse, NY 13210, USA

**Keywords:** Rickettsiae, Ehrlichiae, tick-borne disease, tick-borne disease surveillance, ixodid ticks, *Amblyomma americanum*, *Amblyomma maculatum*, *Dermacentor variabilis*, *Ixodes scapularis*, Texas, USA

## Abstract

Cases of tick-borne diseases, including spotted fever rickettsioses, borreliosis, babesiosis, anaplasmosis and ehrlichiosis, in the United States and territories have more than doubled from 2004 to 2016 and account for 77% of all vector-borne disease reports. In an effort to inform control efforts, the presence of tick-borne pathogens and their vectors was assessed in a recreational park in Walker County, Texas. Here we report data from questing ticks collected on three dates from June 2017 to June 2018. The majority of ticks collected were *Amblyomma americanum* (96.69%) followed by three additional tick species: *Dermacentor variabilis* (2.59%), *Ixodes scapularis* (0.52%), and *A. maculatum* (0.21%). Ticks were pooled and tested for molecular evidence of bacterial and viral pathogens, respectively. All of the 68 pools of *A. americanum* had molecular evidence of the spotted fever group rickettsia, *Rickettsia amblyommatis.* Additionally, six (8.82%) of the *A. americanum* pools contained sequences matching *Ehrlichia chaffeensis*, the pathogen responsible for human monocytotropic ehrlichiosis, and 11 (16.18%) for *E. ewingii*. Three of the *A. americanum* pools demonstrated evidence of *Borrelia lonestari*. The presence of etiologic agents of known human disease in this study merits the continued surveillance efforts of ticks and their pathogens in areas where they could pose risks to public health.

## 1. Introduction

Tens-of-thousands of tick-borne diseases are reported to the CDC by state health departments in the United States annually [1]. Because only a fraction of illnesses are reported, this number does not represent the actual number of cases of tick-borne disease [2]. This under-reporting is unfortunate, as ticks are vectors for a wide array of bacterial, protozoan, and viral pathogens causing such diseases as Lyme borreliosis, spotted fever rickettsioses, anaplasmosis, tularemia, ehrlichioses, babesiosis, and the recently emerging viruses: Heartland (HRTV), Powassan (POWV) and Bourbon (BRBV). Medically important tick vectors are present throughout different geographic areas of Texas, with a steady increase in the presence and geographic expansion of some vectors [2,3,4]; and the incidence of reported tick-borne illness is less than other areas of the United States. Through active surveillance, the relative abundance of ticks and the prevalence of tick-borne pathogens were identified to provide data to target vector and disease control. The most common tick to parasitize humans in the southern United States, *Amblyomma americanum*, transmits a variety of diseases including Heartland virus, ehrlichiosis, tularemia, and rickettsioses. In addition to their competence for pathogen transmission, *A. americanum* has recently been associated with delayed red meat allergy [5,6]. The goal of this project was to establish a site for longitudinal surveillance of *A. americanum* ticks for the presence of tick-borne pathogens. This long-term surveillance will contribute to our understanding of the ecology of tick-borne pathogens in this location and potentially to the region. Here we report our findings from three collections from June 2017 to June 2018.

## 2. Materials and Methods

### 2.1. Tick Collection, Identification and Nucleic Acid Extraction

Ticks were collected under permission of the Texas Parks & Wildlife state park scientific study permit issued to Dr. Donald Bouyer at Huntsville State Park in Walker County, Texas (30°37′42.2″ N, 95°31′33.3″ W). The collection location is a 2083.2-acre recreational park covered by mixed pine–hardwood forest with varying tree density and ground cover and included sampling in the uplands; categorized by an assortment of loblolly pine, red oak, and sweetgum canopy cover, and the lowlands in proximity to a lake where the canopy consists of water oaks, white oaks, blackgum and sweetgum trees. The park wildlife includes white-tailed deer, opossums, moles, skunks, raccoons, eastern gray squirrels and more than 250 species of birds. The weather condition ranges during collections were as follows: 29 June 2017, 31.1–33.9 °C, 49–57% RH; 5 October 2017, 28.3–28.9 °C, 40–44% RH; and 5 June 2018, 30.6–33.3 °C, 49–59% RH.

Three conventional methods were used for collection of questing ticks: dry ice trapping, dragging, and flagging [7,8,9,10]. Flags were swept across substrate and through vegetation, while drags were pulled behind or alongside and sampled at 5 m intervals. Carbon dioxide (CO_2_) traps (20–30 traps per collection) consisted of dry ice placed in a fabric bundle to reduce rapid sublimation atop a 0.5 m^2^ white cloth or inside a Styrofoam container centered on a tape-laden plywood board and were set for 2–3 h. Flagging was also implemented at the CO_2_ trap sites after the traps were collected to harvest ticks in-route or questing nearby. Collections were made on 29 June 2017; 5 October 2017; and 5 June 2018. The collected ticks were identified by morphological means and were surface decontaminated with 3% bleach, followed by 70% ethanol, and then rinsed with sterile water [11,12,13]. In addition to morphological identification, the species of nymphal ticks were confirmed by PCR amplification and sequencing approximately 460 bp from the 3′ end of the mitochondrial 16S rRNA gene (rDNA) using primers 16S+1 (5′-CTGCTCAATGATTTTTTAAATTGCTGTGG-3′) and 16S-1 (5′-CCGGTCTGAACTCAGATCAAGT-3′) [14]. Ticks were then sagittally bisected and separated into pools by species, sex, life stage, and collection site. Half of the ticks were stored at −80 °C for future analysis such as pathogen isolation. Adults were grouped into pools of up to ten tick halves, and nymphs were grouped into pools of up to 25 tick halves. Pools were homogenized with a Retsch MM300 mixer mill (Retsch GmBH, Haan, Germany) for 5 min at 300 Hz in 2 mL microcentrifuge tubes with four volumes of DNA/ RNA Shield (Zymo Research, Irvine, CA, USA) and two 5 mm stainless steel grinding balls. DNA and RNA were co-extracted from half of the homogenate using the ZR Duet DNA/RNA MiniPrep Plus Kit (Zymo Research, Irvine, CA, USA), and the remaining homogenate was stored at −80 °C for further evaluation. The quality and quantity of DNA and RNA were analyzed by either NanoDrop 1000 or Denovix DS-11+ spectrophotometer prior to pathogen detection.

### 2.2. PCR Amplification and RFLP for Tick-borne Bacterial and Rickettsial Detection

Primers ECC (5′-AGAACGAACGCTGGCGGCAAGC-3′) and ECB (5′-CGTATTACCGCGGCTGCTGGCA-3′) were used to screen samples for the 16S ribosomal RNA of Anaplasmataceae [15,16]. Samples which were positive for the initial Anaplasmataceae primer set were then tested with the following primers: HE1 (5′-CAATTGCTTATAACCTTTTGGTTATAAAT-3′) and HE3 (5′-TATAGGTACCGTCATTATCTTCCCTAT-3′) [17] were used for *E. chaffeensis*-specific amplifications while primers EE52 (5′-CGAACAATTCCTAAATAGTCTCTGAC-3′) [18] and HE3 were used for *E. ewingii*-specific amplifications. Primers EphplgroEL(569)F (5′-ATGGTATGCAGTTTGATCGC-3′) and EphgroEL(1142)R (5′-TTGAGTACAGCAACACCACCGGAA-3′), were used to specifically amplify the groEL gene of *Anaplasma phagocytophilum* [19]. Positive samples for *E. chaffeensis* were additionally evaluated with primers targeting the *trp32* gene: FB5A (5′-GTGACATCTTAGTTTAATAGAAC-3′) and FB3A (5′-AAGACTGAAACGTTATAGAG-3′) [20]. Positive controls employed in the respective assays included DNA extracted from *E. chaffeensis* Arkansas strain culture, *A. phagocytophilum* Webster strain culture, and an *E. ewingii* positive tick sample.

Samples were screened for spotted fever group Rickettsia using primers for rickettsial Sca0; Rr190.70p (5′-ATGGCGAATATTTCTCCAAAA-3′) and Rr190.602n (5′-AGTGCAGCATTCGCTCCCCCT-3′) [21]. DNA samples positive for the 533 bp sequence were then categorized using the *Pst*I restriction fragment length polymorphism (RFLP) digest. Enzymatic digestion was performed by incubating 5.0 µL of the amplification products with 1.0 µL of enzyme buffer and 5 U of endonuclease for 1 h at 37 °C, and the digested products were separated on 2% agarose [22]. Additional sequencing of rickettsial *sca5* was performed with primers 120-M59 (5′-CCGCAGGGTTGGTAACTGC-3′) and 120-807 (5′-CCTTTTAGATTACCGCCTAA-3′) [23]. DNA extracted from *R. sibirica* culture was utilized as a positive control.

Nested PCR was used to test samples for the presence of *Borrelia* DNA by amplifying a 330-bp region of the flagellin gene [24]. Briefly, primers FLALL (5′-ACATATTCAGATGCAGACAGAGGT-3′) and FLARL (5′-GCAATCATAGCCATTGCAGATTGT-3′) were used in the primary reaction and 1 µL of primary PCR product was added as a template for the secondary reaction using primers FLALS (5′-AACAGCTGAAGAGCTTGGAATG-3′) and FLARS (5′-CTTTGATCACTTATCATTCTAATAGC-3′). The thermal cycling conditions for both reactions were 95 °C for 3 min, followed by 40 cycles of 95 °C for 1 min, 57 °C for 1 min, and 75 °C for 1 min. DNA extracted from *B. burgdorferi* culture was utilized as a positive control.

Reaction mixes for PCR were prepared in 25 µL reactions using 5PRIME HotMasterMix (Quantabio, Beverly, MA, USA) and 800 µg/mL bovine serum albumin (BSA) water [25]. All PCR thermal cycling conditions used were as previously published unless otherwise noted and resultant products were run on an 1.5% agarose gel stained with ethidium bromide.

### 2.3. Detection of Tick-borne Viruses by Quantitative RT-PCR

The RNA that was isolated from ticks was screened for molecular evidence of Powassan virus (POWV), deer tick virus (DTV), Heartland virus (HTRV), Bourbon virus, and severe fever with thrombocytopenia syndrome virus (SFTSV) by qRT-PCR as described previously with minor modifications [26,27,28,29,30]. Briefly, a SYBR green qRT-PCR assay was utilized with primers designed to be specific to the NS5 coding region of POWV using primers POWV_F (5′-CCGAGCCAAAGTGAGGATGT-3′) and POWV_R (5′-TCTTTTGCCGAGCTCCACTT-3′), and for the detection of DTV using primers DTV-F1 (5′-GTGCCAAGTTTGAATGCGAGGAAG-3′) and DTV-R1 (5′-GAACGGGGCCCAGCGAGAGTGAC-3′) [26]. HTRV was screened for by use of the HTRV-1 Probe (5′-/56-FAM/TGGATGCCTATTCCCTTTGGCAA/36-TAMSp/-3′) with HTRV Primer 1 (5′-CACTGATTCCACAGGCAGAT-3′) and Primer 2 (5′-CCTTTGGTCCACATTGATTG-3′) [27]. The presence of BRBV was tested with use of the BRBV PB1 S2 Probe (5′-/56-FAM/ACCCTTGCTGCATCTTCCACCA/36-TAMSp/-3′) with primer 1 (5′- AACCGAAGGACCATTGCTAC-3′) and primer 2 (5′-ACAGGGACTCCAGAACTTGG-3′) [28]. SFTSV RNA was screened for using SFTSV S gene probe (5′-/56-FAM/TTCTGTCTT/ZEN/GCTGGCTCCGCGC/3IABkFQ/-3′) and primer set (5′-GGGTCCCTGAAGGAGTTGTAAA-3′, 5′-TGCCTTCACCAAGACTATCAATGT-3′) [29]. Standard curves were generated based on previously published methods [30]. Briefly, RNA was extracted from duplicate viral stocks, obtained from the WRCEVA collection at UTMB, using the QIAamp Viral RNA Mini Kit (Qiagen; Germantown, MD, USA) in accordance with the kit instructions. The resulting RNA was serially diluted, and five microliter aliquots of each dilution were mixed, in duplicate, with the corresponding primer and the components from the iTaq Universal SYBR Green One-Step kit (Bio-Rad; Hercules, CA, USA). The efficiency of the RT-PCR for all the primers used in this study was between 98% and 99% (R^2^ value between 0.98 to 0.99).

### 2.4. Nucleotide Sequencing and Analysis

Amplicons were purified with ExoSAP-IT (Applied Biosystems, Waltham, MA, USA) and sequenced using a 3130/3130xl Genetic Analyzer (Applied Biosystems, Waltham, MA, USA) according to the manufacturer’s protocol. Resultant sequences were aligned using Lasergene 12 Seqman Pro software (DNASTAR, Madison, WI, USA) and compared with published gene sequences available in GenBank. Representative samples were triple sequenced and resultant sequences were aligned and submitted to GenBank. Accession numbers obtained in this study are listed in supplementary Table S1.

The minimum infection rate (MIR), was calculated as the ratio of the number of positive pools to the total number of ticks tested per species.

## 3. Results

From three collections in Walker County, Texas, 966 ticks were collected on three sampling dates from June 2017 to June 2018. The majority of the ticks (83.9%) were acquired during the June collections rather than the October collection. Four tick species were identified: *Amblyomma americanum* (96.69%), *Dermacentor variabilis* (2.59%), *Ixodes scapularis* (0.52%), and *A. maculatum* (0.21%) (Table 1).

The most frequent bacterial sequence detected showed 100% identity to *Rickettsia amblyommatis sca0* (Genbank accession nos. MF188914.1, MF188912.1, MF188911.1, CP015012.1, CP012420.1, CP003334.1). Additional sequencing confirmed *R. amblyommatis* through 100% identity of rickettsial *htrA* (Genbank accession nos. CP015012.1, CP012420.1, CP003334.1), and 99.88% *sca5* identity (Genbank accesstion nos. CP015012.1, CP003334.1). *R. amblyommatis* was detected in 100% of *A. americanum* pools tested and additionally, 50% of *A. maculatum* ticks (n = 2) (Table 2). Two additional rickettsial sequences were identified from *Ixodes scapularis* ticks with 99.8% identity and 99.43% identity to the *sca0* of *Ixodes scapularis* endosymbionts (Genbank accession nos. EF689737.1 and XM_002401667.1, respectively).

Six of the *A. americanum* pools contained DNA with 100% 16s rRNA identity to *Ehrlichia chaffeensis* (Genbank accession no. NR_074500.2). Sequencing of a portion of the *trp32* gene from these samples matched with different *E. chaffeensis* strains with half of the samples with 98.3–99.8% identity (Genbank accession nos. CP007477.1, CP007476.1. CP007475.1) and half with 99.8% identity (Genbank accession no. AY307324.1). The prevalence of *A. americanum* pools positive for two sequences of *E. chaffeensis* was 8.82%, whereas the minimum infection rate (MIR) was 0.64%. Eleven pools of *A. americanum* ticks contained DNA with 100% identity to *E. ewingii* 16S rRNA sequences (Genbank accession nos. MF893272.1, U96436.1, NR_044747.1). The percentage of *A. americanum* pools with *E. ewingii* positive sequences was 16.18% with a 1.18% MIR. We observed similar molecular evidence for presence of Ehrlichiae regardless of *A. americanum* lifestage and/or sex (Table 3).

The MIR of *Borrelia lonestari* in the *A. americanum* pools was 0.32%. DNA sequences from three (4.41%) of the pools shared 99.82%–100% identity to the *flaB* gene of a *B. lonestari* isolate (Genbank accession no. AY850063.1). One adult female and one adult male pool had molecular evidence of *B. lonestari* from the June 2017 collection, and one nymph pool from the October 2017 collection, however no evidence of *B. lonestari* was found in the June 2018 collection (Table 3).

None of the tick pool samples tested had molecular evidence of POWV, DTV, HRTV, BRBV, SFTSV or *Anaplasma phagocytophilum*.

## 4. Discussion

In recent years, public health entities have been somewhat unprepared for the emergence of vector-borne diseases such as Zika virus, Bourbon virus, and typhus. Although concentrated efforts by health officials have allowed scientists and clinicians to meet these challenges, the emergence and re-emergence of these pathogens have placed emphasis on the need for increased vigilance for vector-borne diseases. The primary objective of this study was to establish a site for longitudinal surveillance of the pathogen burden of ticks in a park with human recreational activity.

Our data indicate the presence of known bacterial pathogens as well as bacteria of unknown human consequence in a Texas state park. We report molecular evidence of two agents of human ehrlichiosis—*E. ewingii*, the etiologic agent of human ehrlichiosis ewingii, and *E. chaffeensis*, the causative agent of human monocytotropic ehrlichiosis (HME). The first evidence of *E. ewingii* in *A. americanum* ticks in Texas was reported in 2004 [31]. Ehrlichioses are likely vastly underdiagnosed and underrepresented as an important cause of febrile illness, particularly in the southeast and south-central U.S. Furthermore, the disease can be severe (HME has a case fatality rate of 1.9% and hospitalization rate of 49%) [32]. Although often neglected, these data reinforce the importance of considering ehrlichial pathogens as a cause of tick-borne illness in Texas.

The molecular evidence of *B. lonestari* in *A. americanum* ticks is currently of indeterminate significance to human health. The bite of *A. americanum* ticks, which do not transmit *B. burgdorferi*, has been associated with southern tick associated rash illness (STARI). STARI is characterized by erythema migrans and can be accompanied by fatigue, fever, and headache. The etiologic agent of STARI had been hypothesized to be *B. lonestari*, however further research has not supported this hypothesis [33,34]. Until its significance can be resolved, continued reporting of the detection of *B. lonestari* is of value.

Prospective data have shown that the expanding range and presence of *A. americanum* was associated with the increased incidence and decreased severity of reported cases of SFG rickettsioses [4]. The changing epidemiology of SFG rickettsioses in the United States is thought to be influenced by the high prevalence of *R. amblyommatis*-infected *A. americanum* ticks. When bitten by such ticks, a subclinical infection resulting in spotted fever group seroconversion is thought to occur. As documented in *A. americanum* from other regions, *R. amblyommatis* was prevalent in the ticks of Walker County.

Although there was no molecular evidence of any of the emerging viruses in the ticks tested in this study, this establishes a baseline for continued surveillance we are conducting at this location.

## 5. Conclusions

In this study, we successfully collected four species of human-biting ticks using three traditional methods employed for questing tick collection. We will employ these three methods going forward as they will provide a diverse cross-section of the tick species in the sampling area. Molecular testing of these ticks using well-established PCR primers and techniques provided evidence for human bacterial pathogens, however implementing a validated multiplex pathogen assay would be ideal. This would allow us to screen ticks individually with greater efficiency and sensitivity.

These findings stress the importance of longitudinal monitoring in areas such as state parks with coincidental peaks of tick abundance and human recreational activity during warm weather months.

## Figures and Tables

**Table 1:** Number of questing ticks by species, sex, and life stage collected from a state park in Walker County, Texas, USA.

Collection Date	Species	Life Stage/Sex	Total Number
29 June 2017	*A. americanum*	nymph	190
		female	115
		male	116
	*A. maculatum*	male	1
	*D. variabilis*	female	6
		male	10
5 October 2017	*A. americanum*	nymph	150
		female	1
		male	2
	*I. scapularis*	female	2
		male	1
5 June 2018	*A. americanum*	nymph	175
		female	93
		male	92
	*A. maculatum*	male	1
	*D. variabilis*	female	3
		male	6
	*I. scapularis*	nymph	1
		male	1

**Table 2:** Number of positive pools with positive bacterial DNA sequences identified through sequencing of questing ticks from a state park in Walker County, Texas, USA.

Tick Species	Total Pools	Pools with Positive Bacterial Sequences				
		*Borrelia*	*Ehrlichia*		*Rickettsia*	
		*lonestari*	*chaffeensis*	*ewingii*	*amblyommatis*	endosymbiont
*Amblyomma americanum*	68	3	6	11	68	0
*A. maculatum*	2	0	0	0	1	0
*Dermacentor variabilis*	6	0	0	0	0	0
*Ixodes scapularis*	4	0	0	0	0	2
Total	80	3	6	11	69	2

**Table 3:** Incidence of *A. americanum* tick pools with molecular evidence of Borreliae, Ehrlichiae, and Rickettsiae in Walker County, Texas, USA by lifestage and/ or sex: adult female, AF; adult male, AM; or nymph, N.

Collection Date	*Borrelia lonestari*			*Ehrlichia chaffeensis*			*Ehrlichia ewingii*			*Rickettsia amblyommatis*		
	AF	AM	N	AF	AM	N	AF	AM	N	AF	AM	N
29 June 2017	1/12	1/12	0/9	1/12	2/12	1/9	1/12	2/12	1/9	12/12	12/12	9/9
5 October 2017	0/1	0/1	1/6	0/1	0/1	1/6	0/1	0/1	2/6	1/1	1/1	6/6
5 June 2018	0/10	0/10	0/7	1/10	0/10	0/7	3/10	2/10	0/7	10/10	10/10	7/7
Total	1/23	1/23	1/22	2/23	2/23	2/22	4/23	4/23	3/22	23/23	23/23	22/22

## Supplementary Materials

The following are available online at https://www.mdpi.com/2075-4450/10/10/315/s1, Table S1: GenBank accession numbers for representative sequences obtained in this study.
