# Supplementary material for: Detection of Rickettsiae, Borreliae, and Ehrlichiae in Ticks Collected from Walker County, Texas, 2017–2018
Source: Insects. 2019 Sep 25;10(10):315. doi: 10.3390/insects10100315 (PMC6836155; doi:10.3390/insects10100315)
Supplement: Supplementary file 1 [file insects-10-00315-s001.pdf]

Table S1. GenBank accession numbers for representative sequences obtained in this study

	Genbank accession numbers				
**Bacterial sequence**	***flaB***	***16S rRNA***	***trp32***	*sca0*	*sca5*
Borrelia lonestari	MN336354, MN336355	-	-	-	-
Ehrlichia chaffeensis	-	MN33635 0	MN336351, MN336352	-	-
Ehrlichia ewingii	-	MN33635 3	-	-	-
Rickettsia amblyommatis	-	-	-	MN33634 8	MN33634 9
